# Foam Rolling or Percussive Massage for Muscle Recovery: Insights into Delayed-Onset Muscle Soreness (DOMS)

**DOI:** 10.3390/jfmk10030249

**Published:** 2025-06-29

**Authors:** Sebastian Szajkowski, Jarosław Pasek, Grzegorz Cieślar

**Affiliations:** 1 Faculty of Medical Sciences, Warsaw Medical Academy of Applied Sciences, 8 Rydygiera St., 01-793 Warszawa, Poland; sebastianszajkowski@wp.pl; 2 Collegium Medicum, Jan Długosz University in Częstochowa, 13/15 Armii Krajowej St., 42-200 Częstochowa, Poland; 3 Department of Internal Medicine, Angiology and Physical Medicine, Faculty of Medical Sciences in Zabrze, Medical University of Silesia in Katowice, 15 Stefana Batorego St., 41-902 Bytom, Poland; cieslar1@o2.pl

**Keywords:** delayed-onset muscle soreness, percussive massage, foam roller, stiffness, myotonometry

## Abstract

**Background:** Pain manifestations as well as increased muscle tone and stiffness noted in the course of delayed-onset muscle soreness (DOMS) are reflected in altered values of the biomechanical and visco-elastic parameters of muscles. This study aimed to compare the effects of soft tissue mobilization with foam rolling and percussive massage on symptoms of DOMS induced by a standardized muscle fatigue protocol. **Methods:** Healthy volunteers (n = 60) were divided into three groups: FR group—foam rolling (n = 20), PM group—percussive massage (n = 20) and CON group—control/passive rest (n = 20). The fatigue protocol for the gastrocnemius muscle was carried out for development of DOMS in subsequent days. Therapeutic procedures were applied to participants for 3 consecutive days. The results of therapy were assessed by means of myotonometry, performed five times (before, three times during the treatment procedure, and after the end of the procedure). **Results:** Foam rolling significantly reduced the onset and duration of increased muscle tone (*p* = 0.006) and stiffness (*p* < 0.001), unlike percussive massage. The control group exhibited higher tone and stiffness after 48 h, at the peak of DOMS-related pain symptoms. Only foam rolling improved elasticity (decrement, *p* < 0.001), while visco-elastic properties (relaxation, creep) varied inversely with tone and stiffness. Foam rolling led to significantly lower stiffness (day 2) and reduced decrement and relaxation (day 4) compared to the control. Neither therapy was more effective than passive rest for pain relief during the observation period. **Conclusions:** Foam rolling and percussive massage accelerate recovery of muscle tone, stiffness, and elasticity after DOMS as compared to passive rest but offer no added benefit for pain relief.

## 1. Introduction

Exercise-induced muscle damage may cause substantial pain and bring about discomfort in the muscular and skeletal system. Such signs are manifested by tenderness on palpation and limited range of motion (ROM). These impairments begin to occur 12 h after intense exercise and peak at 24–72 h after such intense exercise. Microscopic damage to muscle fibers occurs, which activates the nervous system and can lead to increased protective muscle tension. DOMS causes inflammation and fluid accumulation in the interstitial spaces, which in turn increases pressure within the muscle, resulting in greater stiffness and reduced flexibility. The impairment accompanied by the increased pain can lead to a significant negative impact on muscle performance. Significant physiological factors affecting muscle tension comprise pain and microtrauma associated with DOMS. Inflammation occurring during DOMS is a pathological factor determining muscle stiffness. Muscle flexibility, in turn, is determined by tissue hydration and regular physical activity [1,2]. To mobilize soft tissues and elicit analgesic effects in the course of delayed-onset muscle soreness (DOMS), the following methods can be applied: transcutaneous electrical nerve stimulation (TENS), use of kinesio taping, compression garment, sports massage, foam rolling, and percussive massage with the use of handheld devices (i.e., massage guns).

Foam rolling and percussive massage are two popular methods of self-myofascial release (SMR), because the devices applied are portable and easy to use. However, according to data from the literature, their impact on symptoms related to DOMS remains controversial. Moreover, there is still not enough research that directly compares these two methods of self-massage. While conventional massage usually reduces the symptoms of delayed muscle pain and improves muscle flexibility [1], less is known about self-massage equipment and devices. Recently conducted research on the effectiveness of percussive massage in the treatment of DOMS in gastrocnemius muscles yielded contradictory results [2,3]. Studies concerning the mechanisms by which percussive massage can affect muscle regeneration or performance are few and far between. A recent study confirmed that percussive massage can rapidly and significantly increase blood flow in muscle. This study demonstrates that localized vibrations significantly increase blood flow without affecting the heart rate. The authors point out the need for research explaining the role of the local hormonal response (such as that of histamine) [4]. Moreover, recent reports state that percussive massage reduces the intensity of echo in ultrasound examination (resulting in hypoechoic image). This may imply a reduction in the stiffness and viscosity of muscle tissue as well as the presence of loose connective tissue (i.e., a reduction in resistance during movement as a result of reduced friction and better sliding between fascia layers). As a result, perceived stiffness is reduced without the need to change the structural fascia (reduction of thickness), which would otherwise require reconstruction of the tissue structure and which is not observed during reflex reactions related to the use of various forms of massage [5,6]. The above-mentioned mechanisms may explain the observed therapeutic effects associated with the use of percussive massage.

Similarly, the basic mechanisms of foam rolling interaction in reducing functional disturbances in the course of DOMS continue to remain unclear. Foam rolling is used in myofascial release, which is a form of manual soft tissue therapy used to treat somatic dysfunctions, which can lead to pain and limitation of movement. Most studies reveal that the use of foam rolling has the greatest effect on flexibility, which implies an increase in ROM [7]. It has also been suggested that the use of foam rolling, similar to the application of percussive massage, increases blood flow to the affected tissue and promotes better supply of oxygen to the muscles [8].

The influence of foam rolling on the elasticity and stiffness of muscle and connective tissue has only been indirectly examined thus far. This was performed by following changes in swelling by taking measurements of the circumference and cross-sectional area of the affected muscles. It is possible to determine whether swelling effects are due to variations in muscle architecture (i.e., increases in fascicles angles without changes in length) [9]. The fascia is composed of connective tissues, primarily collagen, which enclose and separate muscles. It participates in the biomechanics of the musculotendinous system by transmitting force. Moreover, fascia contains water, and this water is expelled when fascia is compressed, thus affecting stiffness [10]. Furthermore, the reduction in pain perception after the use of foam rolling is caused by the activation of central pain modulation mechanisms through neural inhibition mechanisms, including Melzack and Wall’s gate control theory of pain [11,12].

To sum up, while percussive massage and foam rolling are increasing in popularity, there is still no clear scientific evidence to explain how their mechanical stimuli translate into therapeutic effects for DOMS. The biomechanical functioning of muscles following therapy with a foam rolling and percussive massage was assessed by examining ROM—an increase in which indicates improved flexibility—and by evaluating muscle stiffness, a decrease in which translates into greater strength [13]. Moreover, there is no consensus as to the optimal therapeutic parameters necessary for supporting recovery processes and counteracting DOMS. There are not many studies directly assessing the biomechanical and visco-elastic properties of muscles, the disturbance of which in the course of DOMS and subsequent accelerated normalization should determine the effectiveness of a given form of therapy.

Myotonometry has been successfully used to study the biomechanical and visco-elastic properties of muscles for several years. This is a non-invasive method of quantitative assessment of mechanical (tone, stiffness and elasticity) and visco-elastic properties (relaxation and creep) of muscular and connective tissue, which are disturbed in the course of DOMS. It can thus successfully complement traditional manual palpation by providing more objective data on contractile properties, and it has been extensively described in the literature [14].

The aim of the study reported here was to compare the effects of soft tissue mobilization with the application of foam rolling and percussive massage in people with symptoms of DOMS induced by a standardized muscle fatigue protocol.

## 2. Material and Methods

Sixty healthy volunteers qualified for this study. The subjects met the following inclusion criteria: age between 30 and 40 years, both women and men, an body mass index (BMI) in the range of 18.5–29.9 (kg/m^2^). An additional requirement was that the participants did not regularly engage in sports activities. This study included individuals within a limited age range and specific BMI range to avoid the influence of morphological and biomechanical changes in muscles that occur as age progresses [15]. The criteria for exclusion from the study were as follows: injuries suffered and treated in the 3 months before the study, injuries treated recently, skin injuries as well as unspecified skin lesions at the measurement sites, subjects’ declaration of general fatigue, fever, suffering from chronic diseases, or staying on medication. The participants were required to refrain from physical exertion for 72 h prior to as well as throughout the experiment. It was found that MyotonPRO has excellent inter-observer reliability and inter-day repeatability in measuring the stiffness of the Achilles tendon and medial gastrocnemius muscle (ICC = 0.83–0.98) [16]. The participants could resign from participation in the study at their own request at any time during the experiment.

### 2.1. Study Design

The prospective clinical trial reported here involved 60 healthy volunteers. The study participants were randomly divided into three groups—the FR group, in which foam rolling was applied (n = 20; 10 men/10 women); the PM group, who were subjected to percussive massage (n = 20; 11 men/9 women); and the CON group or control group, who undertook passive rest (n = 20; 11 men/9 women)—by block randomization (1:1:1 allocation ratio) and block sizes of 3, 6, and 9 using Microsoft Excel software 2024 for Windows 11.

**Figure 1:**
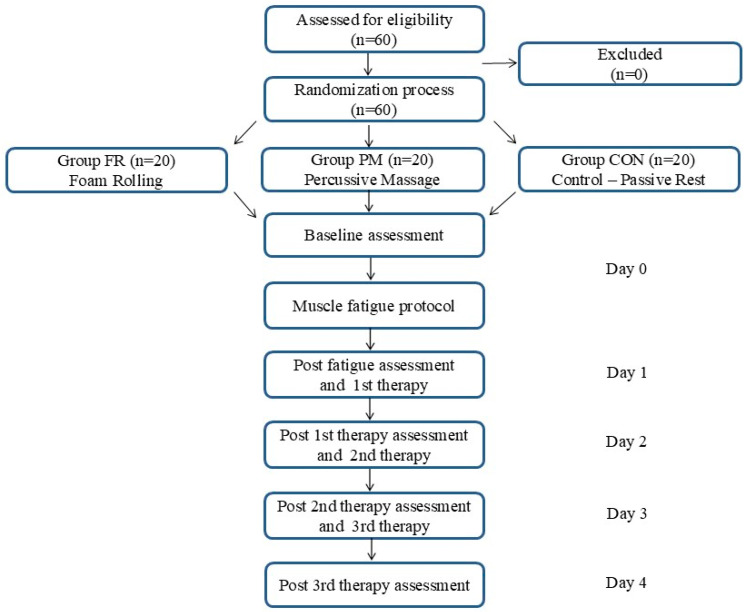
Study design.

The experimental procedures were carried out as follows. On day 0, we undertook measurements by means of a myotonometer and assessment of pain intensity before proceeding with the muscle fatigue protocol. On day 1, we took measurements with the myotonometer and carried out assessment of pain intensity before proceeding with the therapeutic intervention (24 h after the muscle fatigue protocol). On days 2 and 3, we took measurements using the myotonometer and assessed pain intensity before the therapeutic intervention and after the intervention carried out on the previous day. On day 4, we took measurements with the myotonometer and assessed pain intensity. In total, the participants had 3 therapeutic interventions (days 1–3), 5 myotonometric measurements, and 5 assessment procedures concerning pain intensity (day 0–4); (Figure 1). Subsequent examinations and treatment procedures were preceded by 24 h regeneration. Measurements and therapeutic interventions were, in all cases, carried out between 09:00 h and 14:00 h, observing the proper sequence of participants.

Before starting the study, volunteers were asked to fill out and sign consent forms in order to participate in the experiment. During the tests, the same ambient conditions were maintained (air temperature of 22 °C and air humidity of 50%).

### 2.2. Calf Raise—Muscle Fatigue Protocol

Each participant was requested to place the forefoot of the non-dominant limb at the edge of a step and to perform a single leg raise on tiptoes, applying maximum plantar flexion of the ankle joint. Subsequently, the participant was asked to lower the limb to the maximum dorsal flexion, as described in the literature [17,18]. A metronome, set at 1.33 Hz, was used to control the pace of exercise, i.e., for maximum plantar flexion in one stroke followed by four strokes of controlled lowering to maximum dorsal flexion. The study participants were allowed to lean slightly forward, with their fingertips touching the wall just in front of them, in order to maintain balance. The participants had the opportunity to become acquainted with the exercise they were subsequently requested to perform.

The participants performed the exercise continuously until failure. Failure was defined as three consecutive calf raises during which the full ROM was not achieved or during which participants failed to comply with the normal pace set for the exercise. The participants were not aware of the criteria assumed for discontinuing the exercise. A total of 4 series of exercises were performed, with 1 min intermission between series.

### 2.3. Therapeutic Interventions

Foam rolling consisted of 5 min of rolling applied to the entire length of the gastrocnemius muscle. Each roll lasted for 2–3 s. The highest pressure tolerated during the 5 min of foam rolling corresponded to a pain perception level 6 on the numerical rating scale (NRS) [19]. The correct application of the rolling was supervised by an expert with experience in this field. A smooth foam roller was used for therapy.

Percussive massage was applied using a massage gun (Hydragun) with a soft attachment head. The therapy comprised 5 min of self-massage of the entire gastrocnemius muscle. Percussive massage was used at the speed of 53 Hz, or 3200 rpm, as reported previously [20]. Participants were instructed to apply the massage gun to the relaxed calf muscle in a seated position. They were instructed to move the massage gun along the belly of the muscle in a continuous manner, with the vibration speed of the device and the pressure force selected depending on the individual pain tolerance, the desired intensity of which was set at level 6, according to the NRS.

The control group rested passively. Participants in this group did not undergo any therapeutic procedures during the observation period.

### 2.4. Myotonometry

The MyotonPRO device (Myoton, Estonia) utilized in the study was applied to assess the biomechanical parameters of the muscle. It is a digital device consisting of the device body and depth probe (Ø3 mm) that allows non-invasive examination of tissue properties [21]. The myotonometer used in our study utilizes the mechanical dynamic response method, which consists of applying a precise mechanical pulse, registering the dynamic response of the tissue in the form of a signal of physical displacement and acceleration of oscillation, and subsequently calculating the parameters characterizing the biomechanical properties investigated, namely tone [Hz], stiffness [N/m], and decrement (D) [log]. The latter characterizes elasticity; the lower its value, the higher the elasticity and visco-elasticity of the muscle, the relaxation time of mechanical stress [ms], and the ratio of relaxation time to deformation time, which characterizes creep [Deborah number].

The probe was applied perpendicularly to the examined tissue. The device automatically exerted pre-pressure with an application force of 0.18 N, and then, after calibration, the automatically generated 5-fold short mechanical pulse was applied with 0.4 N of force and 15 ms duration, with the aim of deforming the tissue. The applied accelerometer registered the oscillations of the examined tissue [22]. During the test, the coefficient of variation (CV) of each test result was observed; if the CV exceeded 3%, the test was performed once again. All MyotonPRO measurements were taken 3 times and averaged in order to carry out comparative analyses. The total measurement time was up to 20 s.

During the measurements, the subject was placed in a prone position, lying on his stomach with knees straight and feet hanging without support beyond the edge of the couch. The ankle joints were placed in a neutral position. The lower limbs were attached to the table by a strap just above the popliteal fossa. Measurements were taken at a distance of four fingers’ width (approximately 10 cm) below the sulcus popliteus in the thickest most prominent place on the medial head of the gastrocnemius calf muscle, in accordance with a frequently used methodology [23]. Bearing in mind the variability of anatomical structures, the designated place of measurement corresponding to the largest circumference of the calf muscles was additionally verified by means of tape measurements. If required, a necessary correction was made.

All the treatment and measurement procedures were performed by an experienced physiotherapist trained in the field of myotonometry and in conducting scientific research.

### 2.5. Measurement of Pain Intensity

The level of pain intensity was assessed using the numeric rating scale (NRS). This is a pain screening tool, commonly used to assess pain severity at a given moment in time, which utilizes a 0–10 scale, with zero meaning “no pain” and 10 meaning “the worst pain imaginable” [24]. The patients were instructed to score their pain intensity on the NRS scale.

### 2.6. Statistical Analysis

Determination of mean and standard deviation was used to represent the average as well as the typical spread of demographic data. The nature of distribution of the studied variables was tested using the Shapiro–Wilk test. The data turned out not to be normally distributed. The results of all measured variables were presented using median and lower–upper quartiles (Q1–Q3). Friedman’s ANOVA—a repeated-measures analysis of variance by ranks—was used to examine the changes in the examined variables (day 0–4), and a Kruskal–Wallis test by ranks was used to examine the changes between groups (1–3). Post hoc tests with Bonferroni correction were used to analyze the pairwise comparisons. Effect sizes were calculated using partial eta squared (ηp 2) and interpreted according to the following criteria: if 0 ≤ ηp 2 < 0.05, there is no effect; if 0.05 ≤ ηp 2 < 0.26, the effect is minimal; if 0.26 ≤ ηp 2 < 0.64, the effect is moderate; and if ηp 2 ≥ 0.64, the effect is strong [25].

An a priori power analysis was conducted with the G*power software (version 3.1.9.7; Heinrich-Heine-Universität Düsseldorf, Düsseldorf, Germany; (http://www.gpower.hhu.de, assessed on 20 March 2025) [26]. The repeated ANOVA measured within/between interactions with an effect size of at least 0.25, α = 0.05, and 1-β = 0.95. Correlation among repeated measures (0.25) gave a statistical power of 95.1% with a total sample size of 57 subjects. Considering the possibility of withdrawal during the experiment, and wanting to ensure equal group sizes, the sample size was planned to be 60 subjects. All statistical analyses were performed using PQStat 1.8.6. Statistical significance was set at *p* < 0.05.

## 3. Results

The average age in the FR group was 34.31 ± 4.09; in the PM group, it amounted to 33.05 ± 2.96; and in the CON group, it was 34.57 ± 3.44 years of age. The difference was not statistically significant (*p* = 0.904). The average value of BMI was 23.64 ± 2.96 kg/m^2^ in the FR group, 25.05 ± 2.79 kg/m^2^ in the PM group and 24.41 ± 3.02 kg/m^2^ in the CON group. The difference was not statistically significant (*p* = 0.693).

**Figure 2:**
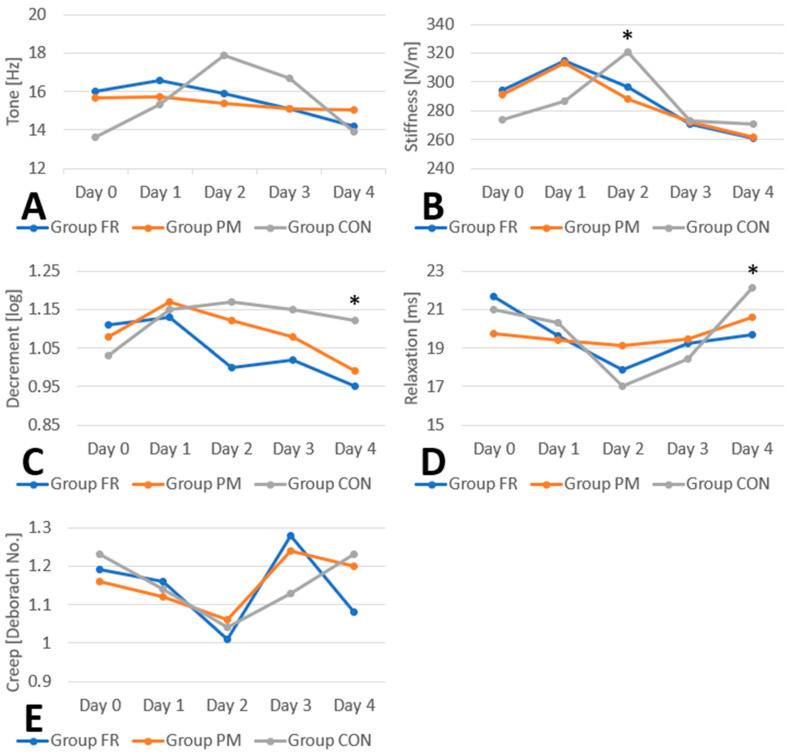
Graphic representation of changes in gastrocnemius muscle (**A**) tone, (**B**) stiffness, (**C**) decrement, (**D**) relaxation, and (**E**) creep from day 1 to 4 in examined groups. Statistically significant differences (*p* < 0.05) between groups are indicated with *.

The results of successive measurements (day 0–4) were compared in each group, and the results obtained in subsequent measurements were compared between groups (FR vs. PM). Statistically significant changes in tone were noted: in the FR group (*p* = 0.006), post-hoc multiple comparisons (Dunn–Bonferroni) revealed statistically significant differences between day 1 and day 4 (*p* = 0.008), and in the CON group (*p* < 0.001), there were statistically significant differences on day 0 vs. day 2 (*p* < 0.001), day 0 vs. day 3 (*p* = 0.013), and day 2 vs. day 4 (*p* < 0.001). No significant differences were observed in the PM group (*p* = 0.086). After the initial increase in tone that was observed in all groups, tone began to decrease earlier, that is, from day 1 in the RF and PM groups. In the CON group, tone began to decrease one day later, from day 2 on, and the noted values turned out to be higher. There were no statistically significant differences in tone between groups on any of the study days (days 0–4) (Table 1A and Figure 2A).

The following statistically significant changes in stiffness were observed. In the FR group (*p* < 0.001), post hoc tests revealed changes on day 1 vs. day 4 (*p* < 0.001) and day 2 vs. day 4 (*p* < 0.001); and in the CON group (*p* < 0.001) on day 0 vs. day 2 (*p* < 0.001) and day 2 vs. day 4 (*p* < 0.001). No significant differences were observed in the PM group (*p* = 0.218). As in the case of tone, stiffness values initially increased in all groups and subsequently decreased earlier, i.e., from day 1 in groups FR and PM and only from day 2 in the CON group. Statistically significant differences in stiffness were noted on day 2 (*p* = 0.042) between the PM group and CON group (in which stiffness values were higher) according to post hoc tests (*p* = 0.040) (Table 1B and Figure 2B).

Upon post hoc testing, values of decrement decreased statistically significantly only in the FR group (*p* < 0.001) on day 0 vs. day 4 (*p* = 0.001) and day 1 vs. day 4 (*p* = 0.001). No significant differences were observed in the PM group (*p* = 0.224) or CON group (*p* = 0.181). As in the case of tone and stiffness, decrement initially increased in all groups and then decreased earlier, i.e., from day 1 in the FR and PM groups and only from day 2 in the CON group. Statistically significant differences in decrement were noted on day 4 (*p* = 0.020); they occurred between the PM group and CON group (in which the values were higher) according to post hoc testing (*p* = 0.005) (Table 1C and Figure 2C).

The values indicating relaxation changed with statistical significance in the FR group (*p* = 0.004), according to post hoc testing, on day 0 vs. day 2 (*p* = 0.013) and in the CON group (*p* = 0.001) on day 0 vs. day 2 (*p* = 0.001) and day 2 vs. day 4 (*p* < 0.001). No significant differences were observed in the PM group (*p* = 0.311). Statistically significant stiffness was noted for day 4 (*p* = 0.011) between the PM group and CON group (*p* = 0.024) and between the FR group and PM group upon post hoc testing (*p* = 0.034); (Table 1D and Figure 2D).

According to post hoc tests, the values of creep changed with statistical significance only in the CON group (*p* < 0.001) on day 0 vs. day 2 (*p* < 0.001) and day 2 vs. day 4 (*p* < 0.001). No significant differences were observed in the FR group (*p* = 0.116) or in the PM group (*p* = 0.324). No significant differences in creep were noted between groups on any day during the study (day 0–4) (Table 1E and Figure 2E).

**Figure 3:**
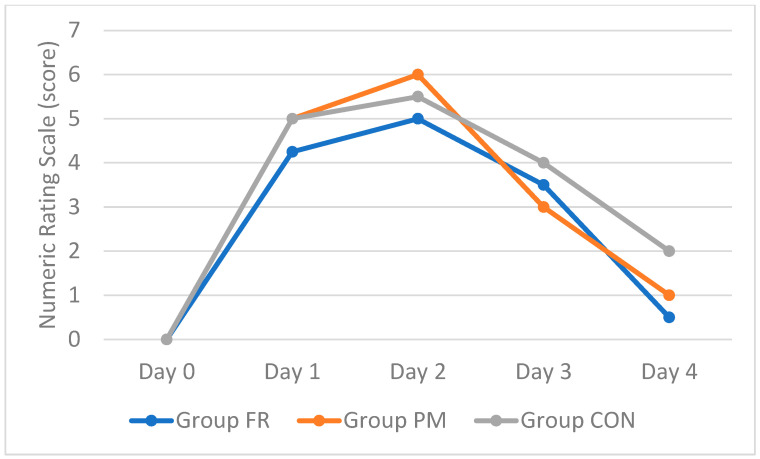
Graphic representation of changes in pain intensity from day 1 to 4 in examined groups.

Statistically significant changes in pain intensity were observed in the FR group (*p* < 0.001), PM group (*p* < 0.001), and CON group (*p* < 0.001). Post hoc multiple comparisons (Dunn–Bonferroni) revealed statistically significant (*p* < 0.001) intensity of pain between day 0 and day 2 and subsequent pain relief between day 2 and day 4 in each study group. There were no significant differences in pain intensity between groups. Statistically significant changes in pain intensity were observed in the FR group (*p* < 0.001), PM group (*p* < 0.001), and CON group (*p* < 0.001). Post hoc multiple comparisons (Dunn–Bonferroni) revealed statistically significant (*p* < 0.001) intensity of pain between day 0 and day 2 and subsequent pain relief between day 2 and day 4 in each study group. No significant differences in pain intensity between groups were noted (Table 2 and Figure 3).

## 4. Discussion

This study evaluated the effects of foam rolling and percussive massage on normalizing biomechanical and visco-elastic muscle parameters altered by DOMS, compared to passive rest. Foam rolling significantly shortened the onset and duration of increased muscle tone and stiffness, while percussive massage showed no significant effect in this respect. The control group exhibited higher tone and stiffness after 48 h, at the peak of DOMS-related pain symptoms. Foam rolling uniquely improved elasticity (decrement), with both therapies showing greater elasticity than passive rest. Visco-elastic properties (relaxation and creep) inversely correlated with tone and stiffness. After foam rolling, significantly lower values compared to the control group were observed for stiffness on day 2 as well as for decrement and relaxation on day 4. Neither therapy surpassed passive rest in pain relief, though pain was highest in the control group at the end of the observation period. Overall, foam rolling and percussive massage accelerated the recovery of muscle properties disrupted by DOMS but did not significantly reduce pain compared to rest.

Foam rolling and massage guns are popular mainly due to recommendations from manufacturers, peers, and personal experience rather than due to scientific evidence. The lack of empirical research and evidence-based guidelines highlights the urgent need for further studies. The results of the study by Michalak B et al. [27] confirm the effectiveness of foam rolling in supporting both immediate and long-term regeneration in cases of therapeutic procedures lasting at least 120 s. The obtained data, as well as the results reported by other researchers, indicate that the type of texture and density of the roller used is of no significance. Different conclusions were reached by Casanova N et al. [9].

The application of foam rolling on the gastrocnemius muscle with constant pressure did not seem to improve recovery after DOMS in the functional properties of plantar flexors, and nor did the gastrocnemius swelling and oxygenation response. However, foam rolling might be useful in acutely increasing the pain pressure threshold after DOMS. The results of a randomized controlled trial indicate that a single session of foam rolling applied on the elbow flexors had no effect on the recovery of maximal isometric voluntary contraction or muscle swelling, ROM, and DOMS [19]. The results of research performed by Sezik AC et al. [28] indicate that significant differences in muscle tone occur when foam rolling is used at different speeds. Slow rolling turned out to show a more significant reduction in muscle tone compared to rapid rolling. Using foam rolling at a speed of 30 rolls per minute along the entire length of the muscle or at an even slower rate can lead to a significant reduction in muscle tone. This has been confirmed by our observations.

Such results may be associated with the slow adaptation of mechanoreceptors present in connective tissue and stimulated by deep pressure [29]. Foam rolling involves the activation of fascial mechanoreceptors, which can lead to a modified proprioceptive signal sent to the central nervous system. This modified signal subsequently regulates the tissue tone [30]. As these receptors become active, motor neuron activity decreases, leading to a reduction in tone. Studies in the existing literature recommend that the duration of rolling be 60–180 s, but the ultimate rolling time may require adaptation to individual needs. In the case of athletes, it most often requires extended time. In a randomized crossover trial [31], the rolling efficiency of the quadriceps femoris muscle was assessed using tissue perfusion (via near-infrared spectroscopy) and stiffness (via tensiomyography and myotonometry).

The data obtained suggest that increased blood flow and altered tissue stiffness may contribute to the effects of foam rolling, although thresholds of statistical significance were not reached. Longer application times may have a greater effect on tissue thixotropy, because rolling can increase tissue temperature through friction and increase hyaluronate fluid pressure through compression.

Increased tissue temperature and fluid pressure can reduce the viscosity of myofascial hyaluronate, resulting in improved tissue elasticity [28]. This may explain the mechanism of the changes we observed in tone, stiffness, and elasticity after self-massage using foam rolling. Another study examined the effects of compression stimulation and free movement during rolling with a foam roller upon ROM and related parameters, from the point of view of the mechanical properties of tissues. It was found that free movement (rolling rate) does not independently affect ROM, while stimulation of pressure independently increases ROM during foam rolling. The combination of stimulation by pressure and free movement may enhance the effect of increasing ROM and may also affect the parameters of biomechanical properties of tissues [32].

The current literature on measuring the therapeutic effects of self-massage using a foam roller continues to expand. The results of an analysis by Cheatham SW et al. [8] suggest that foam rolling may be an effective intervention through which to increase ROM in joints alongside muscle efficiency both before and after exercising. However, despite its popularity, due to the high heterogeneity of the methods used in the research, its physiological effects continue to be studied; currently, there is no consensus on the optimal foam rolling application program.

A systematic search was carried out using the following databases: PUBMED, ISI Web of Science, ScienceDirect, and Cochrane. The search included articles published before September 2023. A total of 25 articles with 517 athletes were studied in depth. The observations found that each study used different exercise protocols, utilizing different treatment methods, application times, and measures, making it difficult to select the results. Due to this heterogeneity of research, it was difficult to draw a conclusion about the correct form and method of foam rolling. However, the analysis of the selected studies indicates that foam rolling seems to have acutely positive effects on flexibility and ROM without affecting muscle performance during activities of maximum strength, favoring perceived recovery and decreasing DOMS [33]. This is also confirmed by our observations.

In general, the main reasons for improved elasticity remain uncertain. Nevertheless, in a sense and from the structural point of view, the positive effects observed can be explained by a temporary and limited connection between fascial tissue and muscle tissue, or by plastic deformation of connective tissue. From a functional point of view, a temporary reduction in pain perception may also lead to improved short-term elasticity [34]. One theory posits that self-myofascial release using a foam roller may elevate skin temperatures and boost blood circulation to the muscle tissue. This increase in blood flow and enhanced muscle tissue warmth may alleviate muscle restrictions and enhance ROM [35].

García-Sillero et al. [2] used a limb-to-limb study design (i.e., a treated vs. control limb) for the gastrocnemius muscle. They concluded that percussive massage followed by four sets of 12 eccentric actions on a flywheel device may help improve muscle recovery by potentially restoring muscle compliance and reducing stiffness measured by tensiomyography.

Leabeater et al. also used a limb-to-limb design with the gastrocnemius muscle and applied a 5-minute-long percussive massage post-exercise (three sets of 20 double-leg calf raises). Their results indicated that the percussive massage applied had no immediate post-exercise effects on muscular performance (ROM, isometric strength, and dynamic endurance) or calf circumference, nor did it significantly affect the perceived muscle soreness immediately, 4, 24, or 48 h post-exercise, in comparison with control in which 20 min passive rest was applied [3].

In our study, we used a relatively short duration of massage gun therapy (5 min). However, it is not known whether the use of massage guns may have a different effect on soft tissues with a longer period of observation. This gap in research is partly filled by the results of our study, where the observation period lasted 4 days, during which participants had three therapeutic sessions with the use of a massage gun. Skinner B et al. investigated the effects of percussive massage on the ROM of the biceps femoris and tissue dynamics by applying myotonometry. Their results suggest that percussive massage can significantly increase the ROM of the biceps femoris muscles by inducing a significant reduction in tissue stiffness levels. The authors reported that changes in tissue tone, elasticity, and relaxation time are connected to an increase in local blood flow. Therefore, if the goal is to increase ROM and reduce the level of tissue stiffness, they recommend using percussive massage [6].

Shear–wave elastography was used to measure tissue stiffness in the deep fascia, muscles, and deep intermuscular fascia by means of shear–wave velocity, as well as the ROM of the ankle joints of volunteers. The measurements were performed before and after 5 min of percussive massage of the medial head of the gastrocnemius muscle. The procedure turned out to modify the stiffness of the deep fascia, and at the same time, it improved the range of dorsiflexion of the ankle. However, the muscles and deep intermuscular fascia did not show any significant changes [36].

Our review shows that foam rolling has stronger scientific support than percussive massage for self-massage. Both methods may help reduce stiffness and improve elasticity, fatigue, and pain from DOMS after resistance training. However, few studies use objective myotonometric or advanced elastography measurements, and most assess the effects of therapy immediately, thus neglecting long-term outcomes. To address this, we evaluated the effects of therapy daily after treatment in order to avoid short-term nervous system reflexes and gather more reliable data.

## 5. Conclusions

Foam rolling and percussive massage have proven to be effective in restoring the correct biomechanical and visco-elastic parameters of muscles, which are subject to changes in the course of DOMS. They reduce the time over which tone and stiffness increase and elasticity is reduced and cause faster normalization of those parameters in comparison with passive rest. Foam rolling has proven more effective in this respect. Foam rolling and percussive massage, however, are not more efficient in alleviating the pain experienced in comparison with passive rest.

## 6. Limitations of the Study

This study had some limitations; the baseline values of the parameters were assumed to be the norm and were merely self-monitored; initial pain sensitivity and the thickness of the subcutaneous fat layer were not assessed [37]; and the body composition of the study participants was not assessed. The gastrocnemius muscle was examined at one point, in accordance with the methods outlined in a previous study [23]. It cannot be excluded that in other areas of the muscle, the measurement results may differ from those reported in this study.

## Figures and Tables

**Table 1:** Changes in (**A**) tone, (**B**) stiffness, (**C**) decrement, (**D**) relaxation, and (**E**) creep of the gastrocnemius muscle (results of the measurements taken by means of MyotonPRO at particular time intervals in examined groups).

	Day 0	Day 1	Day 2	Day 3	Day 4		
	Median (Q1–Q3)					(ηp 2)	** *p*
**(A) Tone [Hz]**							
**FR Group**	16 (17.77–17.2)	16.55 (14.7–17.95)	15.9 (13.8–17.27)	15.1 (13.5–16.35)	14.2 (12.8–15.97)	0.18	0.006
**PM Group**	15.65 (14.75–16.2)	15.75 (15.12–16.58)	15.4 (14.17–16.67)	15.1 (14.12–16.67)	15.04 (14.09–16.47)	0.1	0.086
**CON Group**	13.65 (13.12–15.6)	15.35 (13.47–17.65)	17.9 (15.17–20.57)	16.7 (15.15–19)	13.9 (12.5–17.22)	0.41	<0.001
**(ηp 2)**	0.02	0.01	0.08	0.06	0.02		
* *p*	0.182	0.433	0.058	0.069	0.188		
**(B) Stiffness [N/m]**							
**FR Group**	294.5 (241.25–319.5)	314.5 (270–342)	296.5 (255.25–341.25)	271 (231.5–316)	261 (218.75–312.5)	0.38	<0.001
**PM Group**	291 (240–315.25)	313.5 (267–343)	**288.5** **(244.5–338.5)**	272 (229–312.25)	261.5 (216.25–313.75)	0.12	0.218
**CON Group**	274 (246–351.5)	287 (240.75–358.75)	321 (281.75–368.5)	273 (208.75–317)	270.5 (242–347.5)	0.43	<0.001
**(ηp 2)**	0.01	0.01	0.08	0.13	0.01		
* *p*	0.482	0.630	0.042	0.788	0.423		
**(C) Decrement [log]**							
**FR Group**	1.11 (0.96–1.29)	1.13 (0.96–1.26)	1 (0.85–1.14)	1.02 (0.83–1.13)	0.95 (0.84–1.12)	0.35	<0.001
**PM Group**	1.08 (0.97–1.2)	1.17 (0.97–1.29)	1.12 (0.93–1.21)	1.08 (0.98–1.17)	**0.99** **(0.9–1.03)**	0.07	0.224
**CON Group**	1.03 (0.93–126)	1.15 (0.95–1.2)	1.17 (0.99–1.28)	1.15 (1.03–1.27)	1.12 (0.99–1.33)	0.08	0.181
**(ηp 2)**	0.02	0.01	0.04	0.01	0.1		
* *p*	0.163	0.302	0.103	0.387	0.020		
**(D) Relaxation [m/s]**							
**FR Group**	21.65 (17.35–24.87)	19.65 (15.15–25.17)	17.85 (15.37–24.3)	19.25 (16.12–22.65)	19.7 (15.87–25.47)	0.19	0.004
**PM Group**	19.75 (16.52–20.7)	19.4 (17.57–19.82)	19.15 (18.15–21.12)	19.48 (15.19–22.98)	**20.6** **(18.6–21.9)**	0.14	0.311
**CON Group**	21 (17.8–23.15)	20.3 (14.17–22.4)	17.05 (14.27–19.87)	18.45 (15.4–21.42)	22.1 (18.32–24.5)	0.45	0.001
**(ηp 2)**	0.02	0.02	0.01	0.01	0.12		
* *p*	0.696	0.758	0.260	0.328	0.011		
**(E) Creep [Deborach No.]**							
**FR Group**	1.19 (1.08–1.42)	1.16 (0.98–1.31)	1.01 (0.94–1.3)	1.28 (0.93–1.35)	1.08 (0.95–1.31)	0.09	0.116
**PM Group**	1.16 (0.99–1.34)	1.12 (0.95–1.29)	1.06 (0.95–1.25)	1.24 (0.88–1.29)	1.2 (0.99–1.32)	0.13	0.324
**CON Group**	1.23 (1.04–1.42)	1.14 (0.91–1.34)	1.04 (0.94–1.16)	1.13 (0.98–1.29)	1.23 (1.08–1.47)	0.31	<0.001
**(ηp 2)**	0.01	0.03	0.01	0.01	0.03		
* *p*	0.434	0.871	0.298	0.375	0.143		

* *p*; Kruskal–Wallis ANOVA. ** *p*; Friedman ANOVA. Values that are statistically different from the control group are in bold.

**Table 2:** Assessment of pain at particular time intervals in examined groups.

	Day 0	Day 1	Day 2	Day 3	Day 4		
	Median (Q1–Q3)					(ηp 2)	** *p*
**Numeric Rating Scale (NRS)**							
**FR Group**	0 (0–0)	4.25 (2.75–5)	5 (3–6.5)	3.5 (2–6.25)	0.5 (0.5–1)	0.68	<0.001
**PM Group**	0 (0–0)	5 (4–5)	6 (5–6)	3 (3–4)	1 (0.5–1.5)	0.96	<0.001
**CON Group**	0 (0–0)	5 (4–7)	5.5 (4.25–7)	4 (2.5–6.25)	2 (2–3)	0.47	<0.001
**(ηp 2)**	NA	0.04	0.01	0.01	0.38		
* *p*	NA	0.569	0.680	0.696	0.790		

* *p*; Kruskal–Wallis ANOVA. ** *p*; Friedman ANOVA. NA—not applicable.

## Data Availability

Data are available upon reasonable request to the corresponding author.
